# Odor Sensitivity as a Biomarker of Aging

**DOI:** 10.1093/geroni/igaa057.400

**Published:** 2020-12-16

**Authors:** Marjana Sarker, Scott Leiser

**Affiliations:** 1 University of Michigan Medical School, Ann Arbor, Michigan, United States; 2 University of Michigan, Ann Arbor, Michigan, United States

## Abstract

Recent studies support the deterioration of the sense of smell as an important biomarker for cognitive impairment diseases, including Alzheimer’s disease. The model organism C. elegans has a well-studied olfactory system, which provides an ideal platform to measure loss of smell with aging. The goal of our project is to use the short lifespan and olfactory changes observed in nematodes to identify mechanisms to slow aging and treat age-related diseases. Our approach is to utilize worms at various times of their healthy adult lifespan and to test for their sensitivity to known attractants such as benzaldehyde. These odorants are largely detected by the main AWC olfactory neurons. It is well documented that the responsiveness of AWC decreases with age. Our paradigm is to briefly fast worms to increase motivation before testing their ability to discriminate odors. Our results show that younger worms actively move toward the attractant and show preference for specific attractants. However, older worms frequently do not respond to attractive odors and remain near the point of origin, regardless of motility. These results indicate a decreased odor response with age. Our current work focuses on identifying genes and compounds that positively affect this odor response in older animals. The resulting data can then be tested for their efficacy to improve other aspects of healthspan and potentially longevity.

